# Underweight but not overweight is associated with excess mortality in septic ICU patients

**DOI:** 10.1007/s00508-021-01912-0

**Published:** 2021-09-16

**Authors:** Thomas Danninger, Richard Rezar, Behrooz Mamandipoor, Daniel Dankl, Andreas Koköfer, Christian Jung, Bernhard Wernly, Venet Osmani

**Affiliations:** 1 Department of Anaesthesiology, Perioperative Medicine and Intensive Care Medicine, Paracelsus Medical University of Salzburg, Salzburg, Austria; 2 Department of Cardiology, Intensive Care Medicine & Emergency Department, Paracelsus Medical University of Salzburg, 5020 Salzburg, Austria; 3 Fondazione Bruno Kessler Research Institute, Trento, Italy; 4 Medical Faculty, Division of Cardiology, Pulmonology and Vascular Medicine, University Hospital Düsseldorf, Heinrich-Heine-University Düsseldorf, Düsseldorf, Germany; 5 Center for Public Health and Healthcare Research, Paracelsus Medical University of Salzburg, Salzburg, Austria

**Keywords:** Sepsis, Intensive care, Critically ill, BMI, Obesity paradox, Fluid management

## Abstract

**Background:**

Higher survival has been shown for overweight septic patients compared with normal or underweight patients in the past. This study aimed at investigating the management and outcome of septic ICU patients in different body mass index (BMI) categories in a large multicenter database.

**Methods:**

In total, 16,612 patients of the eICU collaborative research database were included. Baseline characteristics and data on organ support were documented. Multilevel logistic regression analysis was performed to fit three sequential regression models for the binary primary outcome (ICU mortality) to evaluate the impact of the BMI categories: underweight (<18.5 kg/m^2^), normal weight (18.5 to < 25 kg/m^2^), overweight (25 to < 30 kg/m^2^) and obesity (≥ 30 kg/m^2^). Data were adjusted for patient level characteristics (model 2) as well as management strategies (model 3).

**Results:**

Management strategies were similar across BMI categories. Underweight patients evidenced higher rates of ICU mortality. This finding persisted after adjusting in model 2 (aOR 1.54, 95% CI 1.15–2.06; *p* = 0.004) and model 3 (aOR 1.57, 95%CI 1.16–2.12; *p* = 0.003). No differences were found regarding ICU mortality between normal and overweight patients (aOR 0.93, 95%CI 0.81–1.06; *p* = 0.29). Obese patients evidenced a lower risk of ICU mortality compared to normal weight, a finding which persisted across all models (model 2: aOR 0.83, 95%CI 0.69–0.99; *p* = 0.04; model 3: aOR 0.82, 95%CI 0.68–0.98; *p* = 0.03). The protective effect of obesity and the negative effect of underweight were significant in individuals > 65 years only.

**Conclusion:**

In this cohort, underweight was associated with a worse outcome, whereas obese patients evidenced lower mortality. Our analysis thus supports the thesis of the obesity paradox.

## Introduction

Both overfeeding and undernourishment are highly relevant challenges for public healthcare systems worldwide [1]. Obesity is known to be associated with low-grade chronic inflammation and cardiometabolic diseases [2]. It also goes hand in hand with a higher risk for infections and sepsis, which is another common clinical challenge associated with high morbidity and mortality [3–5]. A better understanding of subgroups at higher risk could therefore contribute to improved patient care [6–11]. The pathogenesis and optimal treatment of septic patients is the subject of intensive research, whereas mortality remains high and an often limited functional capacity in surviving patients remains a challenge [6, 12–14]. Underweight is a less common problem than overweight in Western societies. Yet, the relationship between sepsis and underweight is of great interest from a clinical perspective as undernourished septic patients have been consistently observed to suffer from excess mortality [15–17]. On the contrary, the association of sepsis with obesity is complex. While adipose patients have a higher incidence of sepsis, an obesity paradox was observed by different authors [5, 18–20]. This refers to the phenomenon that adipose septic patients, contrary to the experience with many other (chronic) diseases, have a better survival rate than normal or underweight patients [21]. The phenomenon that obesity is a risk factor for the occurrence of a critical illness on the one hand but acutely ill obese patients have a better outcome on the other hand, has also been observed in other clinical situations and remains subject of current debate [22]. Since data on the relationship between obesity and sepsis in particular are contradictory and often based on single center studies, we wanted to investigate the relationship between body mass index (BMI) and mortality in septic patients in a large, multicenter database. The aim of our paper was to evaluate possible associations of overweight and underweight with the outcome of septic patients. We conducted this analysis in the large-scale eICU collaborative research database [23].

### Methods

#### Study subjects

The eICU collaborative research database is a multicenter ICU database, including over 200,000 admissions on 335 ICUs from 208 hospitals across the USA in 2014 and 2015 [23]. We extracted baseline characteristics and organ support data (use of vasopressors and mechanical ventilation) on day one. The database is released under the Health Insurance Portability and Accountability Act (HIPAA) safe harbor provision. Septic patients in this study were identified via the method established by Pollard et al. [23]. Elixhauser Comorbidity Index was calculated using the Agency for Healthcare Research and Quality (AHRQ) method [24, 25].

#### Statistical analysis

We expressed continuous data points as median ± interquartile range and assessed differences between independent groups using Kruskal-Wallis equality of populations rank test accordingly. Categorical data are expressed as numbers (percentage), χ^2^-test was used to calculate univariate differences between groups. The primary exposure was BMI as an ordinal variable based on the World Health Organization BMI classification: underweight (BMI < 18.5 kg/m^2^), normal weight (BMI 18.5 to < 25 kg/m^2^), overweight (BMI 25 to < 30 kg/m^2^) and obesity (BMI ≥ 30 kg/m^2^). The primary outcome was ICU mortality. The secondary outcomes were management strategies, mechanical ventilation, and vasopressor use. We used multilevel logistic regression to fit three sequential regression models for the binary primary outcome to evaluate the impact of the BMI category on ICU mortality. First, a baseline model with the BMI category as a fixed effect and ICU as random effect (model 1) was fitted. Second, to model 1, patient characteristics (age, sequential organ failure assessment, SOFA, score, sex, infection source, ethnics, model 2) were added as independent variables to the model. Third, to model 2, management strategies (model 3) were added to the model. Model 1 and model 2 were used to evaluate the primary and secondary outcomes, whereas model 3 was only used to assess the primary outcomes. We chose the independent variables based on our clinical experience. We calculated adjusted odds ratios (aOR) with respective 95% confidence intervals (95% CI). Additionally, we performed sensitivity analyses, stratifying patients with received fluids > 30 ml/kg/h, creatinine above and below 2.0 mg/dL (arbitrary cut-off), lactate above and below 2.0 mmol/L (arbitrary cut-off), age above and below 65 years (arbitrary cut-off), and SOFA score above and below 10 points (arbitrary cut-off), with and without mechanical ventilation, with and without vasopressor use, and patients with a primary pulmonary focus versus non-pulmonary/all other foci. We performed the stratified sensitivity analyses using model 1. All tests were two-sided, and a *p*-value of < 0.05 was considered statistically significant. Stata/IC 16.1 (StataCorp LLC 2019. Stata Statistical Software: Release 16. College Station, TX, USA) was used for all statistical analyses.

### Results

In total, 16,612 septic patients were included in this analysis, whereas 964 (6%) patients were underweight, 5219 (31%) were of normal weight, 4402 (26%) were overweight and 6027 (36%) were obese. The baseline characteristics are shown in Tables 1 and 2. Obese patients tended to be younger (64 + 19 years vs. 68 + 23 years; *p* = 0.001) but had SOFA scores >10 (14% vs. 12%; *p* < 0.001) more often; however, the most likely clinically relevant differences between the groups were the higher rates of lactate concentrations above 2.0 mmol/L in underweight patients (47% in underweight vs. 44% in normal weight patients) and the higher rates of creatinine concentrations > 2.0 mg/dL in obese patients (36% in obese patients vs. 22% in normal weight patients). Also, we found a significant trend towards higher Elixhauser comorbidity indices in underweight patients compared to all other BMI classes (see Table 1).

**Table 1:** Baseline characteristics in the total cohort stratified for BMI categories

Characteristic	Underweight	Normal weight	Overweight	Obesity	*p*-value
	*n* = 964	*n* = 5219	*n* = 4402	*n* = 6027	
Anthropometric data					
BMI in kg/m^2^, median (IQR)	17 (2)	23 (3)	27 (2)	36 (9)	< 0.001^*^
Age in years, median (IQR)	66 (24)	68 (23)	68 (21)	64 (19)	< 0.001^*^
Age > 65 years, *n* (%)	489 (51)	2,925 (56)	2479 (56)	2814 (47)	< 0.001^*^
Elixhauser Comorbidity Index, median (IQR)	3 (10)	0 (10)	0 (10)	0 (9)	0.003^*^
Risk parameter					
SOFA score points, median (IQR)	5 (5)	6 (5)	5 (5)	6 (6)	0.004^*^
SOFA score > 10 points, *n* (%)	106 (11)	638 (12)	573 (13)	861 (14)	0.002^*^
Heart rate > 110 bpm, *n* (%)	283 (31)	1347 (27)	1145 (28)	1368 (25)	< 0.001^*^
Body temperature > 38 °C, *n* (%)	87 (10)	500 (10)	516 (12)	769 (13)	< 0.001^*^
Biomarkers					
Creatinine mg/dL, median (IQR)	1.0 (1.2)	1.1 (1.3)	1.3 (1.4)	1.5 (1.6)	< 0.001^*^
Creatinine > 2.0 mg/dL, *n* (%)	193 (22)	1226 (25)	1196 (29)	2035 (36)	< 0.001^*^
Lactate mmol/L, median (IQR)	1.9 (2.2)	1.8 (1.9)	1.8 (1.8)	1.8 (1.8)	0.12
Lactate > 2.0 mmol/L, *n* (%)	255 (47)	1358 (44)	1135 (43)	1530 (42)	0.14
Length of stay in h					< 0.001^*^
< 72 h, *n* (%)	610 (63)	3304 (63)	2790 (63)	3657 (61)	–
72–168 h, *n* (%)	257 (27)	1255 (24)	1065 (24)	1524 (25)	–
> 168 h, *n* (%)	97 (10)	660 (13)	547 (12)	846 (14)	–
Fluid management in first 24 h					
Total amount of fluids in ml, median (IQR)	2730 (3316)	2725 (2885)	2684 (2896)	2673 (2881)	< 0.001^*^
Amount of fluid per kg bodyweight in ml, median (IQR)	57 (67)	43 (47)	34 (37)	25 (30)	< 0.001^*^
Amount of fluid per kg BW > 30 ml/kg/h, *n* (%)	322 (71)	1700 (65)	1235 (57)	1253 (42)	< 0.001^*^

*BMI* body mass index, *BW* body weight, *GI* gastrointestinal, *IQR* interquartile range, *SOFA* sepsis-related organ failure assessment

^*^ statistically significant

**Table 2:** Baseline characteristics in the total cohort stratified for BMI categories

Characteristic	Underweight	Normal weight	Overweight	Obesity	*p*-value
	*n* = 964, *n* (%)	*n* = 5219, *n* (%)	*n* = 4402, *n* (%)	*n* = 6027, *n* (%)	
Infectious focus					
UTI	179 (19)	1,162 (22)	1,019 (23)	1,490 (25)	< 0.001^*^
Pulmonary	455 (47)	2,138 (41)	1,600 (36)	2,035 (34)	< 0.001^*^
GI	97 (10)	689 (13)	603 (14)	657 (11)	< 0.001^*^
Cutaneous	56 (6)	320 (6)	320 (7)	718 (12)	< 0.001^*^
Unknown	112 (12)	564 (11)	536 (12)	720 (12)	0.15
Other	62 (6)	337 (7)	308 (7)	384 (6)	0.61
Gynecologic	3 (< 1)	9 (< 1)	16 (< 1)	23 (< 1)	0.20
Ethnicity					
European	720 (75)	4,013 (77)	3,387 (77)	4,816 (80)	< 0.001^*^
African American	142 (15)	552 (11)	434 (10)	616 (10)	< 0.001^*^
Hispanic	35 (4)	223 (4)	197 (5)	185 (3)	0.001^*^
Asian	17 (2)	105 (2)	82 (2)	50 (< 1)	< 0.001^*^
Native American	8 (< 1)	32 (< 1)	36 (< 1)	63 (< 1)	0.10
Other	42 (4)	294 (6)	266 (6)	297 (5)	0.03^*^

*BMI* body mass index; *GI* gastrointestinal; *UTI* urinary tract infection

^*^ statistically significant

The amount of administered i.v. fluid per kg bodyweight was highest in underweight patients and lowest in obese patients (see Table 1; *p* < 0.001). The rates of patients receiving fluids > 30 ml/kg/h in the first 24 h was higher in underweight compared to normal weight and obese patients (72% vs. 65% vs. 42%; *p* < 0.001). We also observed differences in length of stay, with obese patients evidencing the lowest rate of short-term (< 72 h) stays (61% vs. 63% in normal weight patients). The management strategies with respect to vasopressor use and mechanical ventilation were similar across all BMI categories (see Tables 3 and 4 and 5).

**Table 3:** Associations of underweight versus normal weight patients with mortality and management strategies in three multilevel logistic regression models

	Crude events				
Characteristic	Normal weight	Underweight	Model 1^a^	Model 2^b^	Model 3^c^
	*n* (%)	*n* (%)	aOR (95% CI, *p*-value)	aOR (95% CI, *p*-value)	aOR (95% CI, *p*-value)
ICU mortality	553 (11)	134 (14)	1.38 (1.12–1.70, 0.002)	1.54 (1.15–2.06, 0.004)	1.57 (1.16–2.12, 0.003)
Management	–	–	–	–	–
Mechanical ventilation	1175 (23)	221 (23)	1.05 (0.88–1.25, 0.58)	0.83 (0.64–1.08, 0.17)	–
Vasopressor use	1708 (33)	291 (30)	0.87 (0.74–1.02, 0.08)	1.03 (0.82–1.30, 0.80)	–

*aOR* adjusted odds ratio, *BMI* body mass index, *CI* confidence interval, *ICU* intensive care unit, *SOFA* sepsis-related organ failure assessment

^a^Model 1: ICU cluster as random effect

^b^Model 2: Model 1 plus patient level (age, creatinine concentration, ethnics, heart rate, infection focus, lactate concentration, sex, SOFA score)

^c^Model 3: Model 2 plus management strategies (mechanical ventilation, vasopressor use)

**Table 4:** Associations of overweight versus normal weight patients with mortality and management strategies in three multilevel logistic regression models

	Crude events				
Characteristic	Normal weight	Overweight	Model 1^a^	Model 2^b^	Model 3^c^
	*n* (%)	*n* (%)	aOR (95%CI, *p*-value)	aOR (95%CI, *p*-value)	aOR (95%CI, *p*-value)
ICU mortality	553 (11)	437 (10)	0.93 (0.81–1.06, 0.29)	0.88 (0.73–1.05, 0.16)	0.88 (0.73–1.06, 0.18)
Management	–	–	–	–	–
Mechanical ventilation	1175 (23)	935 (21)	0.93 (0.84–1.03, 0.14)	0.96 (0.83–1.11, 0.58)	–
Vasopressor use	1708 (33)	1431 (33)	0.99 (0.90–1.08, 0.79)	0.94 (0.83–1.08, 0.40)	–

*aOR* adjusted odds ratio, *BMI* body mass index, *CI* confidence interval, *ICU* intensive care unit, *SOFA* Sepsis-related organ failure assessment

^a^Model 1: ICU cluster as random effect

^b^Model 2: Model 1 plus patient level (age, creatinine concentration, ethnics, heart rate, infection focus, lactate concentration, sex, SOFA score)

^c^Model 3: Model 2 plus management strategies (mechanical ventilation, vasopressor use)

**Table 5:** Associations of obese versus normal weight patients with mortality and management strategies in three multilevel logistic regression models

	Crude events				
Characteristic	Normal weight	Obese	Model 1^a^	Model 2^b^	Model 3^c^
	*n* (%)	*n* (%)	aOR (95% CI, *p*-value)	aOR (95% CI, *p*-value)	aOR (95% CI, *p*-value)
ICU mortality	553 (11)	552 (9)	0.85 (0.74–0.96, 0.009)	0.83 (0.69–0.99, 0.04)	0.82 (0.68–0.98, 0.03)
Management	–	–	–	–	–
Mechanical ventilation	1175 (23)	1441 (24)	1.08 (0.98–1.89, 0.11)	1.14 (0.99–1.32, 0.06)	–
Vasopressor use	1708 (33)	1977 (33)	0.996 (0.913–1.086, 0.93)	0.97 (0.86–1.10, 0.62)	–

*aOR* adjusted odds ratio, *BMI* body mass index, *CI* confidence interval, *ICU* intensive care unit, *SOFA* Sepsis-related organ failure assessment

^a^Model 1: ICU cluster as random effect

^b^Model 2: Model 1 plus patient level (age, creatinine concentration, ethnics, heart rate, infection focus, lactate concentration, sex, SOFA score)

^c^Model 3: Model 2 plus management strategies (mechanical ventilation, vasopressor use)

Underweight patients evidenced higher rates of ICU mortality (14% vs. 11%; *p* = 0.002), and this finding persisted after adjusting for both patient level characteristics in model 2 as well as in model 3. We did not find differences regarding ICU mortality between normal weight and overweight patients (11% vs. 10%; see Table 4). Obese patients evidenced a lower risk of ICU mortality (9% vs. 11%; *p* < 0.001) compared to normal weight, a finding which persisted after adjustment in all three models (see Table 5).

**Fig. 1:**
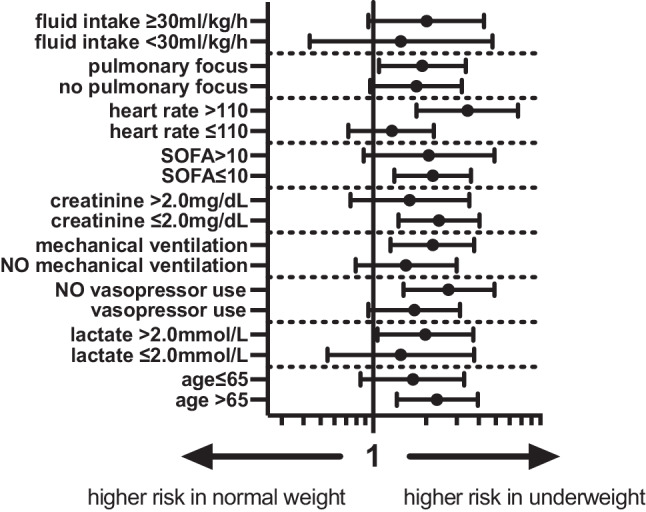
Forest plot of aOR of underweight versus normal weight patients for different subgroups according to model‑1 (aOR 95% CI). *Model 1: ICU cluster as random effect, aOR* adjusted odds ratio, *CI* confidence interval,* SOFA* sepsis-related organ failure assessment

In the stratified analyses for underweight versus normal weight patients, we found a higher risk for ICU mortality in underweight patients above 65 years (aOR 1.52, 95% CI 1.17–1.99), initial lactate concentration above 2.0 mmol/L (aOR 1.41, 95%CI 1.03–1.93), those without vasopressor use (aOR 1.64, 95% CI 1.22–2.22), mechanical ventilation (aOR 1.48, 95% CI 1.12–1.94), initial creatinine < 2.0 mg/dL (aOR 1.54, 95% CI 1.18–2.01), SOFA score < 10 points (aOR 1.48, 95% CI 1.15–1.90), heart rate above 110 bpm (aOR 1.86, 95% CI 1.33–2.59), and those with a pulmonary focus (aOR 1.38, 95% CI 1.04–1.84). The corresponding Forest plot is given in Fig. 1.

**Fig. 2:**
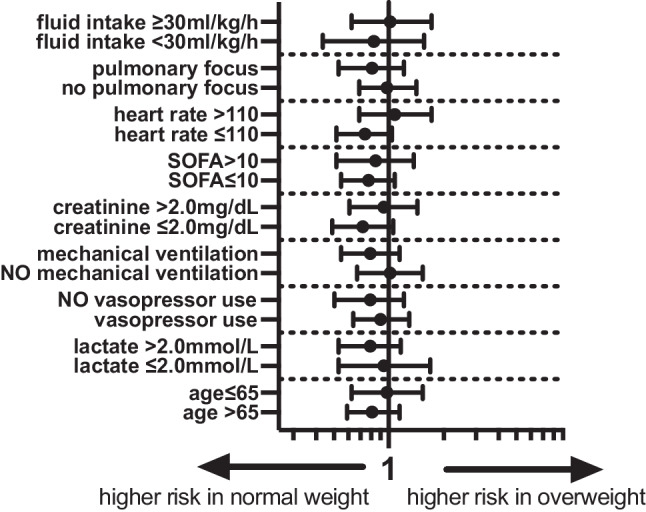
Forest plot of aOR of overweight versus normal weight patients for different subgroups according to model‑1 (aOR 95% CI). *Model 1: ICU cluster as random effect, aOR* adjusted odds ratio, *CI* confidence interval,* SOFA* sepsis-related organ failure assessment

**Fig. 3:**
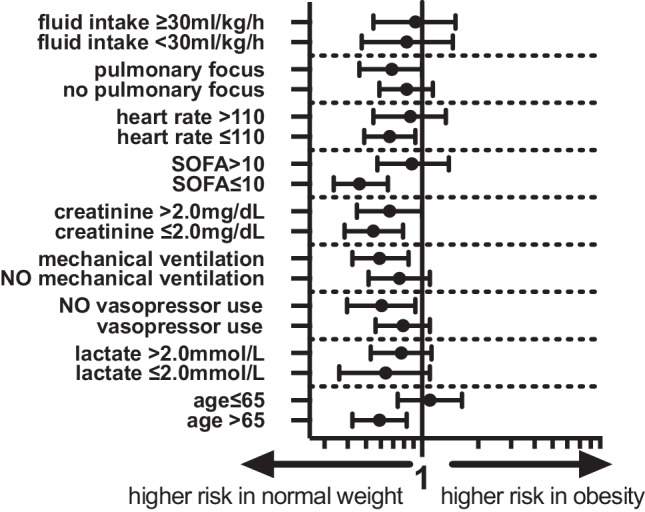
Forest plot of aOR of obese versus normal weight patients for different subgroups according to model‑1 (aOR 95%CI). *Model 1: ICU cluster as random effect, aOR* adjusted odds ratio, *CI* confidence interval,* SOFA* sepsis-related organ failure assessment

In the stratified analyses for overweight versus normal weight patients, we found no differences in the odds of ICU mortality. The corresponding Forest plot is given in Fig. 2. In the stratified analyses for obese versus normal weight patients, we found lower odds of ICU mortality in obese patients in the subgroup of patients aged > 65 years (aOR 0.77, 95% CI 0.65–0.91), patients without vasopressor use (aOR 0.78, 95% CI 0.63–0.96), without mechanical ventilation (aOR 0.77, 95% CI 0.65–0.92), initial creatinine below 2.0 mg/dL (aOR 0.74, 95% CI 0.62–0.89), SOFA score below 10 points (aOR 0.68, 95% CI 0.58–0.81) and heart rate below 110 bpm (aOR 0.82, 95% CI 0.70–0.96). The corresponding Forest plot is given in Fig. 3.

### Discussion

In this study we could not find any differences regarding the use of organ replacement treatment in septic patients with different BMI classes; however, the amount of administered fluid relative to body weight was significantly higher in underweight patients than in normal or overweight individuals. Furthermore, we found an excess mortality of underweight patients, while obese patients showed higher survival rates. These findings could be demonstrated in univariate analysis as well as after extensive adjustment for clinically relevant confounders and were particularly relevant for individuals > 65 years of age. To what extent different fluid management or other factors contributed to the observed robust association of underweight and excess mortality cannot be answered by this study.

The results of our analysis support the thesis of an obesity paradox in septic patients, indicating that obese patients do show higher survival rates compared to normal weight individuals. Similar results were recently shown by Li et al. in another large (*n* = 5563) single center US-based dataset [26]. They could show that not only short-term but also longer-term survival was higher in obese septic patients [26]. This so-called obesity paradox, initially unexpected from the perspective of the clinician (who is used to associate obesity with a higher risk of disease), was also suggested by previous meta-analyses of septic patients stratified for weight classes [20, 27]. It has also been described in patients with acute coronary syndrome or acute respiratory distress syndrome [28, 29]. On the other hand, Jagan et al. were able to show that the effect of BMI on mortality of septic patients in a cohort of 7967 patients was no longer detectable after adjustment for the severity of the acute disease (using acute physiology and chronic health evaluation III score) [30]. In our analysis, however, the effect remained detectable even after extensive adjustment.

The pathophysiological correlate of this observed obesity paradox is beyond the scope of this manuscript, and we can only speculate. A higher BMI was shown to be associated with higher levels of anti-inflammatory interleukins, which could ameliorate the excessive immune reaction, which is typical for sepsis [31]. Also, lipoproteins were shown to have a beneficial effect by binding lipid pathogens and sequestration of lipopolysaccharides (LPS) via (very) low-density lipoprotein receptors [32, 33]; however, other authors have not been able to prove a connection between BMI and the concentrations of cytokines in healthy volunteers [34]. Preclinical basic scientific data on the obesity paradox are also inconsistent [35]. The obesity paradox could have other reasons besides an altered cytokine response [36]. For example, the greater energy storage capacity of obese patients could be beneficial in acute consuming diseases, such as sepsis. Other authors have postulated that the effects of adipose tissue on the renin-angiotensin balance could contribute to better outcomes in obese patients [36, 37].

Nevertheless, despite the relatively robust data, one should remember that the available studies are primarily observational, and thus conclusions on causalities should only be drawn to a limited extent. The premise of the obesity paradox (namely that clinicians associate obesity with higher risk) could contribute to this effect: more obese patients could receive intensive care earlier, be treated more aggressively, and receive more intensive care from nursing staff [38]. Also, the observed effect could be primarily limited to overdominant subgroups, such as older patients, as the patients in many studies had a relatively high average age [19]. This consideration is also supported by our analysis: The protective effect of obesity was only detectable in the subgroup of older patients (> 65 years)—perhaps in this subgroup obesity is an epiphenomenon for e.g. the absence of frailty (which is known to be associated with adverse outcomes), and the possible cardiometabolic risks of obesity are therefore less relevant [39, 40]. On the other hand, these considerations remain speculative in any case, and in our analysis obesity in younger patients was not associated with excess mortality either.

In addition to higher survival rates in obese patients, we have demonstrated excess mortality in underweight patients. This effect is also in line with previous studies on this topic. Zhou et al. were recently able to show that a lower BMI was associated with worse outcome in patients with sepsis [17]. In contrast, a higher incidence of sepsis has been shown for overweight people in a study analyzing over 400,000 individuals [41]. Underweight was shown to be a risk factor in other conditions or after cardiac procedures [42]. As with obesity, the exact causality of the observed association between underweight and the mortality of septic patients is unclear. In contrast to the excess of protective factors in obese patients, it is logical to assume that due to the lack of adipose tissue, and therefore lower binding capacity of pathogenic lipids and LPS mentioned above in the text, underweight patients suffer from a higher mortality in sepsis. It is also known that underweight patients not only have a lean body mass but are frequently undersupplied with essential nutrients, like vitamins and trace elements, usually have less musculature (especially in the old, where it may indicate frailty), can suffer from underweight due to chronic diseases or addiction and often have a lower socioeconomic status [43]. This is also reflected in our work by the significant difference regarding Elixhauser comorbidity indices. The Elixhauser comorbidity index has been validated in its different versions by various authors in the past, controls for comorbidities not directly related to the admission diagnosis, and correlates well with in-hospital mortality. It is a useful marker to summarize multiple pre-existing conditions and to estimate pre-existing morbidity [44]. Furthermore, ethnic factors certainly play a role, as in our study a higher proportion of individuals with European ethnicity was found in obese patients, whereas more Asian patients were observed in the underweight group. Ethnical differences in body tissue composition are well known [45].

An important point would be to provide functional assessments in ICU databases in addition to common intensive care scores in the future. For example, in addition to baseline data such as BMI, it would be important to perform a functional assessment for activities of daily life (ADL, e.g. by Katz index), but also with respect to nutritional status (e.g., by mini nutritional status) and to analyze data accordingly. In the future, this could help to clarify the debate regarding the optimal BMI, since old people are often identified as undernourished despite a normal BMI [46]. Another important point is that this study relies on data from the USA and thus may not be equally applicable to all other countries. For example, the USA remains the high-income country with the highest average BMI, although various countries around the globe follow this trend and Europe also struggles with obesity as a major health challenge [47]. Nevertheless, there are two other important points to consider here: as mentioned earlier, BMI alone is probably not sufficient to fully illuminate the issue, as nutritional status certainly plays a relevant role and here also differences between different countries exist. Another limitation for outcome analyses between distinct countries with currently available databases are different definitions of sepsis and septic shock. In the future, more accurate statements should be possible with newer databases that use the current sepsis‑3 definition [47].

Ward et al. recently demonstrated that suboptimal fluid management could also contribute to this effect, especially in underweight patients [15]. In our study we could detect significant differences in the fluid management of septic patients in the different BMI classes. In the stratified analysis, the excess mortality of underweight patients was also detectable, especially in those patients who received > 30 ml/kg/h of fluids in the first 24 h. On the other hand, the amount of fluid, even in relation to body weight, can only be assessed to a limited extent in isolation from the specific clinical situation. The cut-off of 30 ml/kg/24h is also controversial [48]. In addition, however, the greatest limitation in this respect is the high number of missing values (*n* = 8276) in this variable. We have deliberately decided against imputations, since our clinical experience shows that fluid management is handled very individually. Our finding that underweight patients have excess mortality and also receive a high relative amount of fluid therefore remains descriptive.

We hope that our exploratory analysis of nearly 16,000 septic patients will motivate clinicians to pay special attention to the counterintuitive high-risk group of underweight patients with sepsis. We also hope that our results will help the scientific community to design prospective studies that will shed more light on the impact of BMI in septic patients and optimal fluid management.

### Limitations

This is a retrospective analysis or a dataset which was not a priori set up to investigate the present research question, and our results are therefore limited to thesis-generating character. Although we fitted several regression models and adjusted them for various potential confounding factors, we cannot rule out the possibility that other aspects that may not be included in the data set may ultimately influence the outcome. As discussed above, obese patients might be admitted to critical care earlier compared to other patients, and we cannot adjust for this potential selection bias. Another limitation of our analysis is the lack of long-term data or functional outcomes of the patients. This also limits an analysis regarding the ideal body weight in terms of outcome, as probably functional and nutritional status would be relevant confounders here. Also, data on patient management are not available in greatest detail. We have already commented on the high number of missing values in the data on fluid substitution. We would like to emphasize again that this could lead to a selection bias. Finally, the low mortality in relation to other studies must be stated as a limitation. We have selected the patients using an established method and also observed similar results in the sensitivity analyses for patients with shock (increased lactate, need for vasoactive substances, high SOFA score).

## Conclusion

In this cohort, underweight (BMI < 18.5 kg/m^2^) was associated with a higher mortality in septic patients, whereas a better outcome was observed in obese patients. Our analysis thus supports the thesis of the obesity paradox, and also indicates that in older individuals (> 65 years) BMI could be a surrogate parameter for frailty, as in that age group significant differences regarding outcome were shown. To what extent different fluid management strategies or other factors contributed to the observed robust association of underweight and excess mortality cannot be answered by means of this study.
